# Use of six‐minute walking test to predict peak oxygen consumption in pulmonary vascular disease

**DOI:** 10.1002/pul2.12129

**Published:** 2022-10-01

**Authors:** Rodrigo Torres‐Castro, Elena Gimeno‐Santos, Isabel Blanco

**Affiliations:** ^1^ Department of Pulmonary Medicine, Hospital Clínic University of Barcelona Barcelona Spain; ^2^ Institut d'Investigacions Biomèdiques August Pi i Sunyer (IDIBAPS) Barcelona Spain; ^3^ Department of Physical Therapy, Faculty of Medicine University of Chile Santiago Chile; ^4^ Instituto de Salud Global (ISGlobal) Barcelona Spain; ^5^ Biomedical Research Networking Center on Respiratory Diseases (CIBERES) Madrid Spain

To the Editor,

We read with interest the recent article by Robertson et al.^1^ determining the relationship between 6‐min walk work (6MWW) and the clinical measurements of cardiopulmonary exercise test (CPET) and investigating the ability to predict peak oxygen consumption (VO_2_peak) from 6MWW. To meet their objective, Robertson et al.^1^ retrospectively analysed 63 chronic thromboembolic pulmonary hypertension (PH) and 54 chronic thromboembolic diseases (CTED) patients.

In recent years, exercise capacity has received increasing attention due to its use as a prognostic tool and its association with functional status in patients with pulmonary vascular diseases.^2^ For this reason, both distance walked in the 6‐min walking test (6MWD) and VO_2_peak are included in the risk assessment table of the current clinical guidelines for PH.^2^ However, despite the validity of both tests, the CPET is the gold standard examination for assessing exercise capacity, even though it is an expensive test that requires a skilled operator and specialized equipment with limited availability.^3^

CPET is useful to support diagnosis, permits stratifying patient's risk and serves as a reliable tool to analyse response to treatment.^3^ Despite that, the difficulty of access to this equipment has led numerous researchers to try to create formulas to obtain its main parameter, the VO_2_peak, from other more accessible and easy‐to‐perform tests, such as the 6MWT.^4^, ^5^ Nevertheless, to achieve a reliable, reproducible and useful equation in clinical practice, certain test execution conditions standardized by the scientific societies must be met.^6^, ^7^

Having said that, in the Robertson et al.^1^ study, there are certain aspects of test execution that deserve attention. One of our main concerns is that two different protocols were used for this study to perform the 6MWT. Initially, a 10‐m corridor was used (2015–2019) and later, a 30‐m corridor (2020) knowing that performing the test in different lengths of the corridor has implications on the results. The current ATS/ERS recommendation supports that the 6MWD is very sensitive to variations in methodology, including changes in track layout and length.^6^ Moreover, a recent study showed that the difference between the two protocols could reach almost 70 m apart.^8^ Additionally, multiple studies have attempted to validate a shorter (less than 30 m) course length for the 6MWT, resulting in a significantly smaller distance.^9^, ^10^

The reasons to obtain different distance walked, if the length of the corridor is not the same, are multiple. First, patients slow down the walking speed when going through the cone, which decreases the final distance. For example, a person who walks 600 m will only turn 20 times in the 30‐m corridor, but will turn 60 times in the 10‐m corridor. Additionally, if the patient is an older adult or has balance problems, it will be even more difficult to accelerate in each lap.^11^, ^12^ These reasons have led the ATS/ERS to recommend using a standardized corridor of 30 m or at least 20 m in their clinical guidelines.^6^ Furthermore, given this recommendation dates back to 2002, it is not understandable to use circuits shorter or longer than these standardised distances.^13^

Another important point refers to what the exercise tests actually measure. The CPET is an incremental test that measures maximum or peak exercise capacity, whereas the 6MWT is usually a submaximal test since it is not incremental. Although our group demonstrated that, in patients with PH, this test represents a maximum capacity,^14^ it could not be confirmed that this is the case with all the patients evaluated by Robertson et al.,^1^ since approximately only half of them, showed PH. It is likely the group with CTED, with a mean pulmonary artery pressure of 16 ± 6 mmHg, behaves like the rest of the population and the 6MWT is a submaximal exercise. Furthermore, our data showed that the response to exercise in PH patients indicates cardiovascular limitation due to increased right ventricular afterload. However, in the supplementary material shown by Robertson et al.^1^ notable differences in the hemodynamic profile of both populations can be seen and conclusions are not so clear.

Finally, we believe that it is essential to generate predictors of maximal capacity, particularly because of the high cost and sophisticated equipment the CPET requires, making it challenging to assess maximal exercise capacity in a low‐resource setting. However, the validation of these equations must be very rigorous so that reproducibility is not affected, and could be used with complete confidence in clinical practice.

## AUTHOR CONTRIBUTIONS

**Rodrigo Torres‐Castro**: conceptualization, writing–original draft, writing–review and editing. **Elena Gimeno‐Santos**: conceptualization, writing–original draft, writing–review and editing. **Isabel Blanco**: conceptualization, writing–original draft, writing–review and editing.

## CONFLICT OF INTEREST

The authors declare no conflict of interest.
